# Understanding prokaryotic adaptation through advanced DNA methylation detection techniques

**DOI:** 10.1093/ismejo/wraf143

**Published:** 2025-07-09

**Authors:** Ziming Chen, Chian Teng Ong, Elizabeth M Ross

**Affiliations:** Queensland Alliance for Agriculture and Food Innovation, The University of Queensland, St. Lucia, QLD 4072, Australia; Queensland Alliance for Agriculture and Food Innovation, The University of Queensland, St. Lucia, QLD 4072, Australia; Queensland Alliance for Agriculture and Food Innovation, The University of Queensland, St. Lucia, QLD 4072, Australia

**Keywords:** adaptation, prokaryotes, DNA methylation, long-read sequencing, methylation calling

## Abstract

DNA methylation, a versatile epigenetic modification in prokaryotes, is a crucial regulator of various biological activities, such as genome defence, gene expression, and DNA repair. The most common DNA methylation form in prokaryotes is N6-methyladenine, where a methyl group is added to the adenine. Orphan and restriction-modification system methylases constitute the main methylation systems in prokaryotes. Prokaryotes can adapt to environmental fluctuations through orphan methylase regulation and phase variation of restriction-modification systems, which generate diversified methylomes that modulate the expression of genes. Modern sequencing techniques, including single-molecule real-time sequencing and Nanopore sequencing, enable the characterization of several methylation patterns simultaneously and facilitate the study of prokaryotic epigenomics. This review introduces the prokaryotic DNA methylation systems and prokaryotic adaptation through DNA methylation. Finally, we summarize the current sequencing techniques capable of characterizing methylation forms applicable to prokaryotes and their future perspectives.

## Introduction

**Figure 1:**
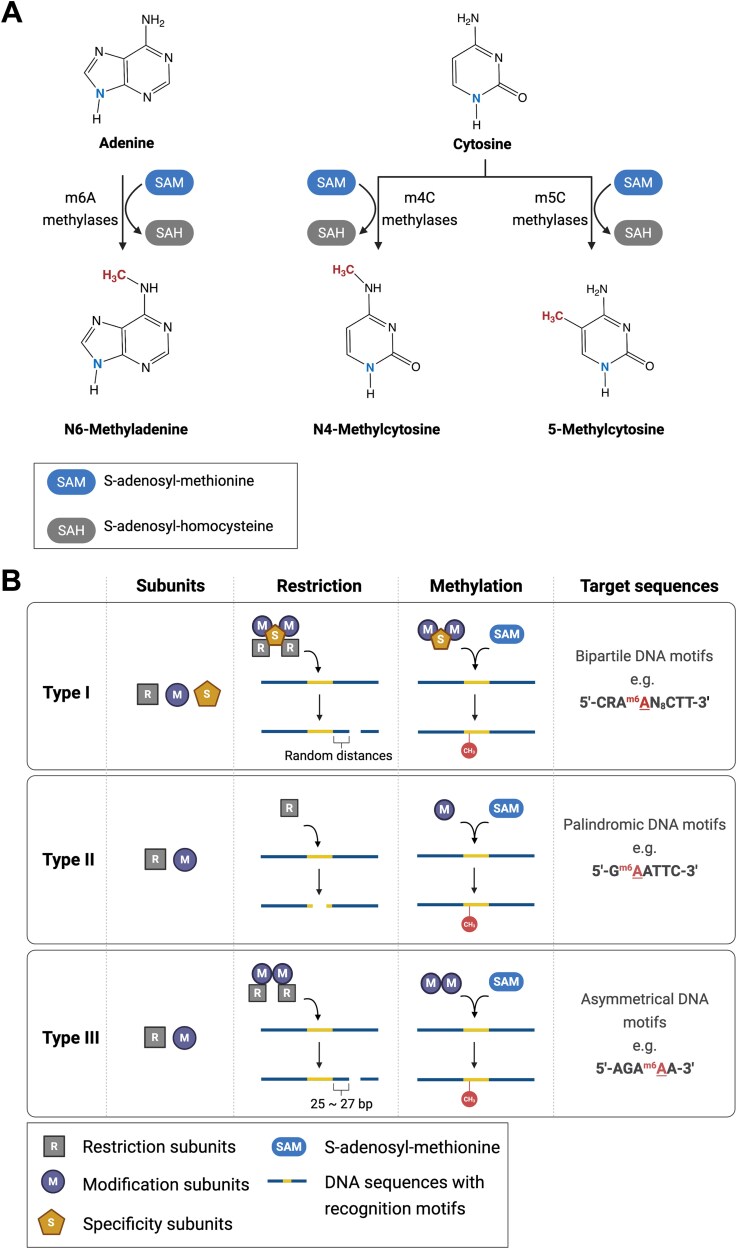
(A) Primary types of DNA modification forms in prokaryotes and (B) types of RM systems. There are three main types of DNA methylation patterns in prokaryotes: m6A, m4C, and m5C. The corresponding methylase in each scenario can transfer the methyl group in S-adenosyl-methionine to targeted bases. Type I, II, and III are three RM systems capable of modifying DNA sequence motifs in prokaryotes. The type I system consists of restriction, modification, and specificity subunits. Modification components methylate the bipartite motifs, while the restriction counterparts recognize the cleavage sites at varying distances from the motifs. The type II system lacks specificity subunits, but restriction and modification components recognize the same motifs, which are usually palindromic. The type III system only contains restriction and modification subunits, but the restriction event occurs at around 25 bp downstream of the asymmetrical motifs.

The term “epigenetics” was originally introduced in 1939 by Conrad Waddington who combined the genetics and developmental biology areas [1]. Currently, epigenetics is the study of heritable variations in gene expression without mutation of DNA sequences. Histone modification, DNA methylation, and non-coding RNA all constitute epigenetic mechanisms. DNA methylation is the biological process where a methyl group is added to DNA facilitated by DNA methylases. When a methyl group is added to the nucleotides in promoter regions, it can downregulate gene expression by either hindering the interaction between DNA and transcription factors or by recruiting relevant gene repressors [2]. In prokaryotes, there are three major types of DNA methylation patterns (Fig. 1A), namely N6-methyladenine (m6A) [3], N4-methylcytosine (m4C) [4], and 5-methylcytosine (m5C) [5]. Among them, m6A is the most prevalent modified form in prokaryotes, followed by m4C [6].

Adaptation to various environmental changes is crucial for the survival and multiplication of organisms. In addition to mutation, reversible adaptation can be achieved through gene regulation by DNA methylation, which makes organisms adaptable to various external factors without permanently changing their genetic material. DNA methylation in prokaryotes is carried out by two principal categories of DNA methylases, known as orphan (solitary) methylases and restriction-modification (RM) system methylases. RM systems are recognized as a host genome defence tool in degrading viral genetic materials lacking specific methylation patterns and gene expression modulation [7–10]. The diversity and phase variation of prokaryotic DNA methylation systems enable global gene regulation and adaptation in a wide range of environments [11, 12], such as soil, ocean, and the digestive tract.

Traditional DNA methylation profiling methods, including Sanger and bisulfite sequencing, were first developed for m5C detection. Although Sanger sequencing can identify DNA methylation through the distinct signal peak heights from the methylated base, its weak signal detection and low throughput limit its wide application [13]. In comparison, bisulfite sequencing has been regarded as the gold standard for m5C detection, but not for other methylation forms [6]. Enzyme-linked immunosorbent assay–based methods are known for their simplicity in methylation detection, while they are only useful for measuring bulk methylation levels. Although mass spectrometry (MS) based methods can detect multiple DNA methylation forms globally, the high cost and time investment limit their wide application [14]. In addition, none of these methods allows the calling of different types of methylomes simultaneously at a single-nucleotide level [6].

Compared with previous DNA methylation characterization approaches, third-generation sequencing techniques, including Nanopore-based sequencing (offered by Oxford Nanopore Technologies, also known as ONT) and single-molecule real-time (SMRT) sequencing (offered by Pacific Biosciences, also known as PacBio), allow the detection of multiple forms of methylation simultaneously, which has recently intensively advanced the study of prokaryotic epigenomes. Furthermore, multiple analysis tools, such as the ONT’s basecaller Dorado and kineticsTools [23], have been developed for Nanopore or SMRT data to identify different methylation patterns. These current sequencing techniques have also facilitated the development of prokaryotic methylation analysis and meta-epigenomics of environmental samples [24, 25]. In this review, we introduce DNA methylation systems relevant to prokaryotic epigenomes, examine how prokaryotic environmental adaptation could be regulated by methylation variation, and summarize the sequencing techniques utilized for epigenetic modification identification.

## Prokaryotic DNA methylation systems

Prokaryotic DNA can be methylated by two major methylase categories: orphan methylases and RM system methylases (Table 1). Orphan or solitary DNA methylases are enzymes functioning in the absence of a cognate restriction enzyme. In contrast, the RM DNA methylases usually function along with the corresponding endonucleases of the RM system (Fig. 1B). While some Type II DNA methylases from the RM system lack cognate restriction components [26], they are still classified into RM DNA methylases in this review. RM systems, one of the prokaryotic genome defence tools, are abundant in prokaryotes. RM genes are present in around 83% of prokaryotic genomes, almost two times higher than the abundance of the other antiviral system, namely clustered regularly interspaced short palindromic repeats–CRISPR-associated proteins (CRISPR-Cas) system [27]. Hence, the methylases from RM systems are more common than those classified as orphan in prokaryotes. These two DNA methylation systems in prokaryotes endow them with diverse methylation patterns.

**Table 1:** Major DNA methylases in prokaryotes.

**DNA methylases (abbreviation)**	**System**	**Representative organism**	**Target sequences** ^**2**^ **(5′-3′)**	**References**
DNA adenine methylase (Dam)	Orphan	*Escherichia coli*	G^m6^ATC	[15,16]
Cell cycle regulated methyltransferase (CcrM)	Orphan	*Caulobacter crescentus*	G ^m6^ANTC	[17]
M.MtuHIII methyltransferase (MamA)	Orphan	*Mycobacterium tuberculosis*	G^m6^AGGTC	[18]
DNA cytosine methyltransferase (Dcm)	Orphan	*E. coli*	C^m5^CWGG	[15, 19]
Leptospiral orphan methylase A (LomA)	Orphan	*Leptospira interrogans*	^m4^ CTAG	[20]
Type I restriction-modification system specificity determinant HsdS_A1_ (HsdS_A1_)^1^	Type I	*Streptococcus pneumoniae*	5’-CRA^m6^AN_8_CTT-3′ 3’-GYTTN_8_G ^m6^AA-5’	[8]
Type I restriction-modification system specificity determinant HsdS_A2_ (HsdS_A2_)^1^	Type I	*S. pneumoniae*	5’-CRA ^m6^AN_9_TTC-3′ 3’-GYTTN_9_ ^m6^AAG-5’	[8]
Type IIG restriction-modification enzyme (Cj0031)	Type II	*Campylobacter jejuni*	5’-CCYG ^m6^A-3’	[12]
Type IIL restriction-modification enzyme MmeI (MmeI)	Type II	*Methylophilus methylotrophus*	5’-TCCR ^m6^AC-3′	[21]
Type III restriction-modification system methyltransferases ModA13 (ModA13)	Type III	*Neisseria gonorrhoeae*	5’-AGA^m6^AA-3′	[22]
Type III restriction-modification system methyltransferases ModP1 (ModP1)	Type III	*Actinobacillus pleuropneumoniae*	5’-CAA^m4^CT-3′	[9]

^1^The DNA methylase (HsdM) in the Type I RM system functions with the specificity subunit (HsdS).

^2^W: A or T; Y: C or T; R: G or A.

### Orphan DNA Methylases

Deoxyadenosine methyltransferase (Dam) is one of the orphan DNA methylases. Existing as a monomer with 31 kDa, Dam was first identified in *Escherichia coli* [15, 28]. Its homologues also exist in multiple gamma-proteobacteria, such as *Haemophilus influenzae* [29]. The adenine of the 5’-GATC-3′ motif is the methylated site of Dam [16]. Dam shows similar efficiency in methylating adenine in motifs under unmethylated or hemi-methylated conditions [30], indicating that Dam functions as both the methylation pattern writer and transmitter. Dam is a highly processive enzyme, capable of methylating 55 of 116 targeted sites before dissociating from a DNA strand [31]. However, the processivity of the Dam can be altered by the flank sequence of the 5’-GATC-3′ recognition motif [32, 33].

Another orphan methylase is cell cycle-regulated DNA methyltransferase (CcrM), which was originally identified in *Caulobacter crescentus* and methylates the adenine of the sequence 5’-GANTC-3′ [17]. CcrM homologues are prevalent in several alpha subdivisions of the Proteobacteria, such as *Agrobacterium tumefaciens* [34]. A previous study reported CcrM methylated adenine in a processive manner [35]. However, this conclusion was challenged by a subsequent study and confirmed that CcrM methylated targeted sites in a distributive way [36], which means that CcrM dissociates from the DNA strand after each methylation. In addition, CcrM shows a preference for hemi-methylated DNA with a 1.5-fold methylation rate, compared to unmodified DNA [36].

DNA cytosine methyltransferase (Dcm) is also an orphan methylase isolated from *E. coli* [15]. Dcm shows a preference for the methylation of the second cytosine of the 5’-CCWGG-3′ motif (W refers to A or T) [19]. In addition, the cytosine of this motif is also the recognition site for methylases in the EcoRII RM system [37], indicating the potential role of Dcm in the modulation of this system. Furthermore, a study found that a range of Dcm methylases are homologues of the methylases in the EcoRII RM system after the phylogenetic analysis [38].

### Methylase-based RM systems

RM systems are considered both host genome defence and gene regulation tools in prokaryotes. Generally, RM systems are classified into four major categories (I, II, III and IV), according to their compositions and cleavage sites (Fig. 1B). Of these, I, II, and III are methylase-based, while IV only possesses endonucleases (outside the scope of this review). Restriction (R), modification (M), and specificity (S) subunits, with corresponding encoded genes namely *hsdR*, *hsdM*, and *hsdS*, constitute the enzymatic complex (M_2_S or R_2_M_2_S_1_) in the Type I RM system. Two target recognition domains (TRDs), namely N-TRD and C-TRD, in the specificity subunit each account for half of the specificity for recognizing the bipartite DNA motif, which is separated by random nucleotides in a particular sequence [39]. Upon binding through specificity elements, the modification subunits (methylases) can modify the unmethylated target nucleotides of both strands at the recognition sequences. Studies indicated that the cleavage of DNA occurred only when the translocation of the RM enzymatic complex on DNA is hindered by different factors, such as supercoiled DNA and collision with other complexes [40]. Hydrolysis of DNA is exerted by restriction components (endonucleases) [39] at random distances from recognition sites, and it is asynchronous with the binding event [41]. Here, although the modification complex M_2_S can work without endonucleases in some cases [39], Type I RM system methylases (HsdM) are still classified as the RM systems in this review.

Type II systems, the most abundant RM system [27], are characterized by the independent activity of methylase and endonuclease during the host genome protection process. Specificity subunits are absent in this system, while both methylases and endonucleases share the same recognition motifs. Most endonucleases hydrolyze unmethylated DNA by identifying the palindromic sequences with 4–8 bp length and produce “sticky” or “blunt” ends, which also makes them a useful tool in DNA recombination and fragmentation analysis [42]. Depending on the subtypes of methylase in this system, they can either methylate a single strand or both strands at specific sites [39], which is similar to the cleavage pattern of corresponding endonucleases [42].

Type III systems consist of *res* and *mod* genes, the expression of which forms an enzymatic complex (M_2_ or R_2_M_2_) with restriction and methylation enzymes. The restriction event of the Type III system relies on the modification components due to the absence of specificity activity in the restriction subunits. The recognition sites of modification enzymes are characterized as the unmethylated asymmetrical DNA sequences of 5–6 bp length [43]. In addition, similar to the Type I system, the hydration of DNA of the Type III system occurs at the defined position 25–27 bp downstream from the recognition motifs and requires the recognition of two inversely oriented motifs [44, 45]. However, unlike the cleavage that occurs in both strands, the methylases only modify one DNA strand due to the asymmetrical recognition [43, 45].

The RM and orphan methylation systems in prokaryotes involve a variety of methylases targeting different motifs. These diversified methylated motifs increase the complexity of gene networks in prokaryotes. Therefore, the in-depth study of these systems allows us to identify the importance of genetic modifications in the prokaryotic lifecycle, including gene regulation, adaptation, and the maintenance of lineage stability.

## Adaptation under epigenetic control

In nature, the occurrence of DNA methylation in organisms can be genetically determined, spontaneous, or environmentally induced [46]. The existence of methylation in genetic materials could be short-term and unstably inheritable, which is characterized by the reconversion to their original status after several generations or the removal of environmental stress [47, 48]. On the other hand, some methylated patterns can be long-term, ranging from their maintenance across somatic progenies [49] to the parental imprinted genes in mammals [50]. Hence, DNA methylation may function independently of DNA sequence changes in Darwinian evolution [51] and serve as a reversible and plastic function in the life cycle of an organism.

Long-term methylomes in organisms can be determined by genetic factors. Methylation quantitative trait loci (mQTL) refer to differential methylation levels resulting from single nucleotide polymorphisms (SNPs). In eukaryotes, SNPs have shown effects on some methylation patterns of different tissue samples [52, 53]. Furthermore, the methylomes of several diseases are associated with the SNP effects, such as psychiatric disease [54]. These SNP-methylation interactions may suggest that long-term methylation memory can be stored in the genome and the epigenetic patterns can be stably inherited along lineages.

Organisms can adapt to environments through DNA methylation. Unlike mutations that directly change the genetic sequences, DNA methylation keeps genetic sequences relatively intact while modulating gene expression, supporting organisms to live in various ecosystems. For instance, a study proposed the latent role of DNA methylation in seahorses during their transition from pelagic to benthonic habitat [55]. In eukaryotes, the methylation in promoters, enhancers, and gene bodies can enhance or depress gene expression [56]. Generally, the presence of methylated bases can sterically impede the interaction between DNA and transcription factors [2, 57]. On the other hand, the methylated motifs can also recruit gene repression proteins competing with transcription factors for binding at these specific sites [2, 57]. Additionally, the introduced methyl group can alter the surface electrostatics of the DNA strand, disrupting transcription factors, and repressors binding [58]. These interferences can lead to differential gene expression levels, and eventually generate diverse phenotypes [2]. In prokaryotes, the methylation of promoters also affects the expression of nearby genes, while gene bodies with different methylated states are also potential transcriptional regulators [59]. However, the mechanism of how methylation enhances transcription levels remains mostly unknown.

### Orphan DNA Methylases in prokaryotic adaptation

One of the classic models for gene regulation of orphan methylases is based on Dam, in which Dam methylase alters the methylation patterns through the competition for binding of specific motifs with other proteins, such as transcription factors. For instance, under low leucine-responsive regulatory protein (LrP) levels in *E. coli*, Dam completely interacts with the 5’-GATC-3′ motif within the *pyelonephritis-associated pili (pap)* promoter followed by methylation, hindering the subsequent binding of LrP [60]. Likewise, the interaction order of oxidative stress regulator (OxyR) and Dam at *antigen 43 (agn43)* promoter also leads to interruption of *agn43* expression [61]. In these cases, the expressions of *pap* and *ant43* are crucial for the adhesion and biofilm formation of *E. coli,* respectively. In addition, gene expression can be affected by the flanking sequences of methylase recognition motifs as well. For instance, a study found the flank sequences of 5’-GATC-3′ motifs in the promoter of *pap* impede the processivity of Dam in *E. coli*, contributing to its binding competition with LrP and distributive methylation [32, 33].

The orphan methylase Dam allows the adaptation of *Salmonella enterica* under bacteriophage stress by altering the methylation pattern on the *opvAB* operon. During the phase invasion, the bacteriophages utilize the lipopolysaccharide O-antigens on the *S. enterica* membrane as receptors. However, the *opvAB* operon in *S. enterica* can express two inner membrane proteins, OpvA and OpvB, to increase the resistance to bacteriophage [62, 63]. For example, under the phage stress, the OpvA and OpvB were expressed when the first and third 5’-GATC-3′ motifs in the *opvAB* regulatory region were methylated via Dam orphan methylase [48]. The structure of lipopolysaccharide O-antigens was significantly changed due to the expression of OpvA and OpvB, which reduced the phage attachment and eventually increased their resistance to phages; however, this also reduced the *S. enterica* virulence [62]. In contrast, upon removing the phage stress, the *opvAB* operon was reversibly switched back to OFF status through the methylation of the second and fourth 5’-GATC-3′ motifs in the *opvAB* regulatory region, resulting in the increased virulence of *S. enterica* [48, 62].

Another example of orphan methylase regulated adaptation is related to *Mycobacterium tuberculosis*, a pathogenic bacterium for tuberculosis. Despite being a strictly aerobic species, its infection process involves several environments with a range of oxygen concentrations in hosts. A study found that the presence of M.MtuHIII methyltransferase (MamA) could alter the methylation patterns of the *M. tuberculosis* genome under hypoxic conditions [18]. These results indicated that MamA potentially regulated gene expression to support the survival of *M. tuberculosis* in different microenvironments [18]. However, the expression of some genes, such as *Rv0142* and *CorA*, can also be interrupted by other factors without MamA, suggesting a further study was needed to validate whether the increased transcription levels of these genes during oxidative stress were related to methylation from MamA [18]. Overall, these studies indicate that methylation in prokaryotes can be a response to environmental stimuli, potentially allowing the prokaryotes to adapt to the stimuli through gene regulation with tradeoff strategies.

### Phase variation in prokaryotic adaptation

**Figure 2:**
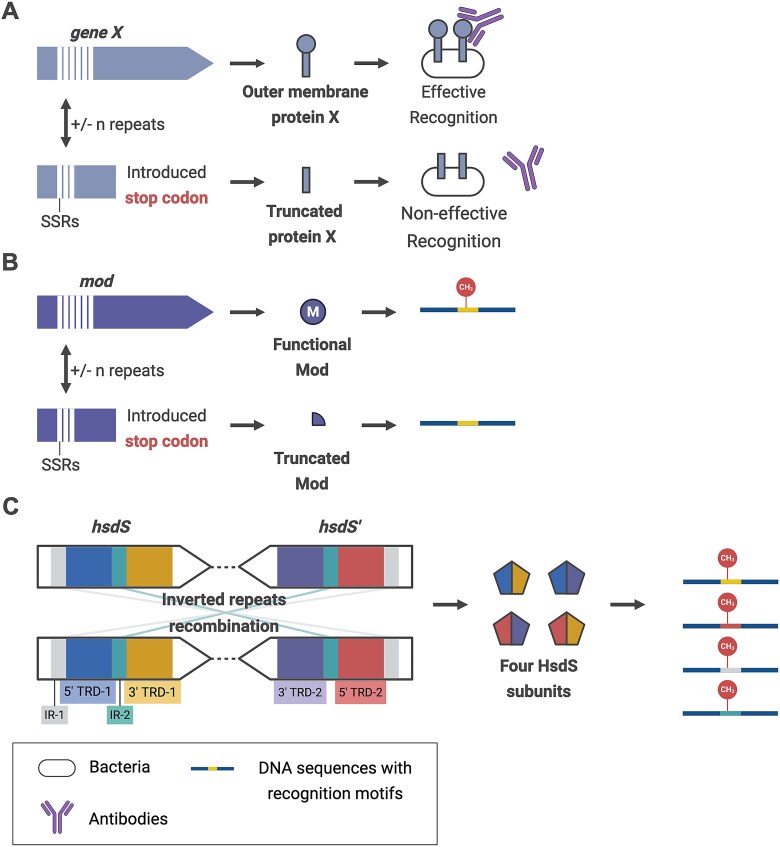
Phase variation in prokaryotes. (**A**) The dynamic numbers of SSR in the protein-coding gene regulate gene expression. The truncated protein structure leads to recognition failure by antibodies. (**B**) The varying SSR numbers enable the modulation of gene expression switching. Truncated mod cannot methylate corresponding motifs. (**C**) The recombination of IRs allows the expression of four different HsdS subunits, which can modify four different motifs. SSRs, simple sequence repeats; IRs, inverted repeats.

The initial biological understanding of methylases from the RM systems was their involvement in the immune system, protecting the host genome from viral invasion. However, studies of phase variation of RM systems have revealed their role in prokaryotic gene regulation [7, 9, 10, 64]. Phase variation is a reversible and random ON/OFF switch control for gene expression. The genetic framework that modulates the expression of numerous genes is called phase-variable regulon or phasevarion [7]. The occurrence of phase variation of genes related to RM systems generates diverse methylation patterns in prokaryotes, which not only endows them with a repertoire of immune responses against diverse phages but also the capacity to accommodate a variety of environments [65]. Furthermore, phase variation can also occur in different types of genes, such as membrane surface protein genes (e.g. *sclB*) and regulatory protein genes (e.g. *mga*) (Fig. 2A) [66]. This review focuses on the phase variation in RM systems.

### Phase variation in type I RM system

In the Type I RM system, methylation site shifting can be achieved through the formation of a novel specificity by recombining inverted repeats (IRs) in different *hsdS* genes (Fig. 2C), or by the recombination of recognition domains from various specificity subunits [40]. Inverted repeats are DNA motifs, followed by their reverse complement sequences downstream on the same strand. In addition to IR-related phase variation, a recent study found that almost 10% of the evolved Type I RM systems can undergo variation through the expansion/contraction of simple sequence repeats (SSRs; repeated DNA short sequences with several nucleotides in tandem), similar to the other two RM systems [12, 67].

There are multiple examples of gene regulation of phase variation in the Type I RM System. The phase-variable Type I methylase was first reported in *Mycoplasma pulmonis* [68], where distinct specificities generated from recombining inverted repeats were identified. However, the relationship between these phase-variable methylases and phenotypic alteration was not analyzed [68]. A further study found that the *hsdS_A1_* allele in OFF status due to IR recombination, which can transform *Streptococcus pneumoniae* colonies from opaque to transparent and increase their adhesive ability to epithelial cells [8]. In another experiment, a similar transformation of colony phenotype was observed [69], but the reduced adherence in *hsdS_A_-*expressing variants was not observed. Meanwhile, *hsdS_A_*-expressing variants showed degraded survival performance in mature biofilms due to IR recombination [69]. In addition to the investigation of HsdS phase variation, direct knock-out of the *hsdM* gene resulted in significantly increased expression of redox pathways and drug-related genes, such as *katG* and *gyrA*, stimulating drug resistance of *M. tuberculosis* [64]. Likewise, the knockout of the Type I specificity gene (HPP12_0797) in *Helicobacter pylori* caused the methylation loss in the target motifs 5′-GAAN_8_TAG-3′ and gene expression changes, such as the Chaperone and heat shock proteins [70].

### Phase variation in type II RM system

Understanding of Type II system phase variation is limited compared to Type I and Type III RM systems. Here, although the function of Type II methylases works independently of the endonucleases, they are not classified as orphan methylases in this review. In Type II methylase genes, the expansion/contraction of SSRs can initiate phase variation by introducing stop codons (Fig. 2B). This results in the translation of inactive methylases and, therefore, the lack of methylation in certain prokaryotic genome regions [71]. Previously, the switching status of the Type IIG methylase gene (*cj0031*), controlled by the ployC/G repeat number within the open reading frame, was first identified in a food-borne pathogen *Campylobacter jejuni* [72]. A subsequent study confirmed that the ON/OFF of *cj0031* can alter the methylation patterns as well as some phenotypes, such as invasion and biofilm formation [12]. However, it remains unknown whether Type IIG methylase modulates gene regulation through the methylation of the promoter or transcription region [12]. In addition to Type I system phase variation, *H. pylori* also harbours phase-variable Type II DNA methylase, M.HpyAIV, with poly(A) repeats within the intergenic region, where the loss of an adenine generated the inactive M.HpyAIV [73]. Furthermore, the knockout of the M.HpyAIV gene resulted in lower expression of catalase [73].

### Phase variation in type III RM system

Recently, many studies have focused on the phenotypic changes resulting from phase variation of the Type III RM system. SSRs exist in around 18% of the open reading frames of the Type III *mod* genes [74], suggesting their likelihood of causing phase variation in Type III methylase genes (Fig. 2B). Numerous studies demonstrated the gene regulation by SSR-related phase variation in the Type III RM system. For example, the phase-variable *mod* gene in the *H. influenzae* Type III system is due to the expansion/contraction of 5’-AGTC-3′ tetranucleotide repeats [75]. This was initially observed from *H. influenzae* colonies with two distinct colours after transforming with synthesized plasmids containing a *mod* gene with different repeats fused in the *lacZ* open reading frame [75]. SSR-regulated phasevarions in the Type III RM system of *H. influenzae* were subsequently proposed [7], where the switching of the *mod* gene by 5’-AGTC-3’ SSR expansion/contraction altered the expression of heat-shock protein genes, such as *htpG* and *dnaKJ*, therefore resulting in two distinct phenotypes. In another study, a significantly higher cellular growth rate and larger biofilm mass were observed in *Actinobacillus pleuropneumoniae* when the methylase gene, *modP1,* was switched on with 10 SSRs [9]. Furthermore, *A. pleuropneumoniae* expressing the other methylase gene, *modP2,* with 9 SSRs showed increased resistance to diverse antibiotics compared to *A. pleuropneumoniae* with the *modP2* gene turned off [9].

Orphan methylases and the phase variation in RM methylase produce abundant methylation motifs in prokaryotes, impacting downstream gene networks and their global expression. This global regulation facilitates the adaptability of prokaryotes in different environments. While research on archaeal adaptation regulated by DNA methylation is limited, studies examining the DNA modification system in archaea [24, 26, 27] have identified several archaeal methylases homologous to the bacterial methylases, indicating their potential gene regulation roles in archaea. Overall, gene regulation by DNA methylation enables prokaryotes to achieve the optimal physiology to manage external pressures under different environments.

## Advanced detection techniques of prokaryotic DNA methylation

### Third-generation sequencing techniques for methylation characterization

Previously, DNA methylation could be characterized by identifying peak signal deviations, such as Sanger sequencing [13], or by the enzymatic/chemical conversion of modified nucleotides to other nucleotides followed by sequencing, such as bisulfite sequencing [76–78]. However, these methods are either low throughput, laborious, or limited to one type of modification. Currently, SMRT sequencing [79] and Nanopore sequencing [80] are two third-generation sequencing techniques capable of detecting multiple methylation forms simultaneously in a genome. Compared with previous sequencing methods, the amplification process is not mandatory in these two technologies, which allows the retention of methylation information. Furthermore, Nanopore-based methylome data from the ONT platform is not affected by methylated RNA, which can generate false-positive signals [81].

In SMRT sequencing, inter-pulse duration (IPD) is the value of the time interval (or the pulse fluorescence) between each incorporation of a nucleotide during sequencing, which is the measurement of the polymerase kinetics. It has been demonstrated that polymerase kinetics can be affected by the primary and secondary structures of DNA [82]. Hence, SMRT can identify various methylomes through distinct IPD signals resulting from the methylation modification [79]. SMRT has different detection sensitivities for different methylated bases because of their different signal-to-noise ratios, with higher sensitivity to m6A and m4C but lower to m5C [18, 79]. However, it showed that the conversion of m5C to 5caC by TET1 enzymes or a higher sequencing coverage can provide improved signals of m5C in SMRT [83, 84]. Likewise, for 5-hydroxymethylcytosine (5hmC) detection, the chemical tags on 5hmC not only generate a stronger kinetic signal but also reduce the requirement for a higher sequencing coverage [85]. In Nanopore sequencing, each base generates a unique current disruption as it passes through the nanopore in the flow cell. These distinct electric current patterns were utilized to identify different bases and to discriminate different methylation forms, which overcomes the m5C detection difficulty and complexity in the SMRT sequencing [80, 86, 87].

**Figure 3:**
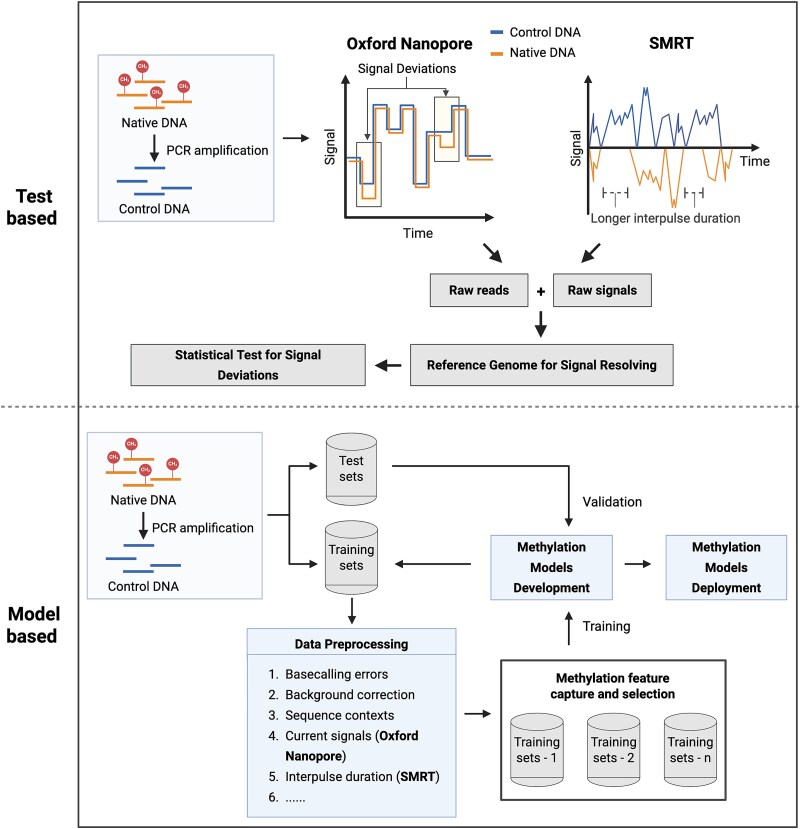
Methylation characterization methods for SMRT sequencing and Nanopore data. The development of both methods requires native and control samples. Native samples retain all DNA methylation information in the DNA, while control samples are generated from native samples by PCR amplification, where all DNA methylation information is removed. During sequencing, the methyl groups in the DNA base generate distinct electrical currents or inter-pulse duration signals. In the test-based approaches, the reads are aligned to the reference genome to resolve the raw signal, followed by the identification of DNA methylated positions through a statistical test between native and control samples. In the model-based strategy, both training and validation datasets contain native and control reads. Before the model training using the training dataset, the data are preprocessed based on the sequenced context and other factors. After the training, the validation dataset is used to evaluate the quality of the model. Once the model is developed, control samples are unnecessary in the model-based approach.

Generally, both SMRT and Nanopore sequencing identify modified DNA bases using test-based or model-based approaches (Fig. 3). The test-based method relies on statistics between the control and methylated samples [88–90]. The methylated samples (native) are DNA with intact methylation information, while the control samples (negative) are usually generated from PCR amplification, where all DNA methylation is wiped out. For test-based approaches, data are first aligned to the reference genome to map signals to specific genomic positions, which can resolve the raw signal and facilitate the subsequent identification of DNA methylation positions. In SMRT sequencing, the modified positions in the genome were identified by comparing the IPD signal variations between the native and control samples using statistical tests, such as the log-likelihood ratio (LLR) statistic [84, 88] and the Two-Sample t-test [90]. In Nanopore-based methylation calling, the electrical current signal deviations between the methylated and unmethylated groups are used to identify modified DNA bases. To statistically analyse these differences, tests such as Mann–Whitney U-test [91] and Kolmogorov–Smirnov test [92] are used. These statistical test-based methods avoid the dependency on large training datasets with different genomic sequence contexts and have the potential to uncover the whole collection of modified nucleotides in the genome. However, this approach also increases the cost and workload of generating the unmethylated data.

Trained methylation model methods employ deep learning or machine learning to predict the modified base in the genome. Generally, the DNA methylation model development includes training and validation processes for both SMRT and Nanopore-based systems. Both training and validation datasets involve native and control reads from multiple species with different sequence contexts. The sequence and signal features from native and control reads are fed into the model for training with various algorithms, such as Convolutional Neural Network [23, 93] and Random Forest [94], which allows the estimation of the methylation probability of a specific position. Once the model is trained, the validation dataset (not including the training dataset) is used to evaluate the quality of the model. Although control samples are still required for the development of the model, they are not necessary for users during the analysis, allowing easier DNA modification detection compared to the statistical test-based approaches, at the potential cost of some accuracy. In addition, using a convolutional neural network for model training improved m5C identification in an SMRT dataset without enzymatic conversion of DNA samples [95], illustrating the usefulness of the model-based methods. However, the model-based methods may not be generalizable to other DNA methylation features if they are not defined in the training dataset.

Several third-party packages have been developed for m6A identification for SMRT data, such as iDNA-MS [96] and CNN6mA [94]. However, they were mostly trained based on eukaryotes and can only support the m6A prediction with reference species provided in the software, which limits their usage in prokaryotic epigenomics. On a positive note, the kineticsTools in SMRT Link software has been a tool for the classic standard workflow of m6A characterization for SMRT data since late 2011 [23], which provides test-based and model-based methylation detection options. However, a reference genome is still required in the kineticsTools for the methylation calling.

Currently, only a few of the packages can detect m6A modifications in the data produced by ONT’s Nanopore system, and most of them cannot predict the methylation sites without a prior model training dataset. For instance, some packages, such as DeepMP [93], can only conduct m6A analysis on prokaryotes with known methylated patterns, such as 5′-GATC-3′, and a complete reference genome, which limits the applicability in most systems. Two packages, namely NanoMod [92] and nanodisco [97], are now available for researchers to perform de novo methylation detection on prokaryotic DNA samples without the prior knowledge of preferred methylated motifs of the organisms, but control samples and reference genomes are still necessary for the methylation analysis. Encouragingly, ONT released the m6A detection model for the basecaller Dorado on 19^th^ May 2023, which can provide m6A detection at the single-nucleotide level without the necessity of negative control samples and reference genomes.

### Discrepancies and challenges in third-generation sequencing techniques for methylation calling

The introduction of third-generation sequencing techniques has accelerated the characterization of prokaryotic DNA methylation. However, some controversial results have been documented when using Nanopore and SMRT sequencing approaches. A study showed that the m5C measured in *Yarrowia lipolytica* using immunoassay was not detectable when applying the Nanopore methylation analytical tool modPhred [81]. Likewise, a study also demonstrated that the m5C signal from TET SMRT was lower than the one measured by antibodies [98]. Some recent studies also found that several methylation motifs characterized by the SMRT protocol were not detectable in Nanopore data [9, 97]. These findings indicated that different techniques can introduce variation. Thus, a consistent method for methylation analysis is recommended.

The analytical tools (Table 2) developed to detect m6A exhibit variable performance, with some tools excelling in speed and precision but detecting fewer modification sites, while others provide higher accuracy in specific datasets. When comparing these tools, it is important to understand the definitions of accuracy and precision. Accuracy is the distance between observed and true values, while precision refers to the level of proximity of measured values. For instance, a study conducted a benchmark analysis of existing m6A tools for Nanopore data, including DeepSignal, Megalodon, and DeepMP [93]. They found that DeepMP had the highest accuracy and precision in predicting m6A in 5′-GATC-3′ motifs of the pUC19 dataset [93]. However, the m6A within 5’-GATC-3′ is only a fraction of prokaryotic methylation; a large amount of m6A is from other motifs. In addition to Nanopore, another study benchmarked the existing tools m6A models for SMRT data, including iDNA-MS, CNN6mA, and Deep6mA [94]. They found CNN6mA showed the second highest m6A prediction accuracy in *Xanthomonas oryzae pv. Oryzicola (Xoc) BLS256*, a γ-Proteobacteria, compared to the other models. However, in terms of precision, CNN6mA only ranked third among the others in *Xoc. BLS256*. Therefore, given these differences, there is still a demand for developing software with high accuracy and wide detection ranges.

**Table 2:** Packages for m6A detection using third-generation sequencing data.

Packages	Methods	Methylation patterns	Reference genomes	Detection levels	Negative controls	Systems	References
nanodisco	Multi-label classification framework	m5C, m6A, m4C	Yes	Motif discovery	Yes	ONT, R9.4	[97]
Megalodon^1^	Recurrent neural network	m5C, 5hmC, m6A	Yes	Base level	No	ONT, R9.4.1	NA
Dorado	Neural network	m5C, 5hmC, m4C, m6A	No^2^	Base level	No	ONT, R10.4.1	NA
Hammerhead	Strand-specific error pattern metric	NA^3^	Yes	Base level	No	ONT, R10.4.1	[99]
kineticsTools	Convolution neural network or Two-Sample t-Test	m5C, 5hmC, m4C, m6A	Yes	Base level	Depends on models	SMRT^4^	[23]
iDNA-MS	Random Forest algorithm	5hmC, m4C, m6A	Yes	Base level	No	SMRT^4^	[96]
CNN6mA	Convolutional neural network	m6A	Yes	Base level	Yes	SMRT^4^	[94]

^1^Megalodon has since been deprecated by ONT.

^2^Reference genomes are only used for mapping after methylation calling.

^3^Hammerhead can only distinguish whether the base is modified, but cannot identify the specific modification forms.

^4^No sequencing chemistry is specified.

The detection of methylation in Nanopore data is still challenging because several factors need to be considered, including basecalling errors [93], intrinsic noise [100] and sequence contexts [101]. In addition, the rapid advancements in sequencing chemistry and basecalling methods result in a high turnover rate, with frequent updates, improvements and replacements [102]. Often, previously developed packages based on outdated sequencing techniques may not be compatible with the data generated from new Nanopore models. The rapid sequencing development and complexity of methylation calling present a large software engineering challenge and could lead to the cessation of software maintenance. On the other hand, the low sensitivity to m5C in SMRT has increased the difficulty in m5C characterization in samples. There is an urgent demand for developing software to characterize m5C in SMRT data without enzymatic or chemical conversion before sequencing, despite its low prevalence in prokaryotes [95]. In a nutshell, the fast turnover rate of sequencing chemistry, basecalling methods, and technical limitations, combined with the lack of consistent development upgrades in existing tools, have introduced new challenges for accurate methylation calling from third-generation sequencing data.

### Prokaryotic DNA methylation advancements in third-generation sequencing techniques

Although DNA methylation characterization by third-generation sequencing technology is still developing, its versatility and efficiency have facilitated the in-depth analysis of prokaryotic DNA methylation. For instance, by using Nanopore sequencing, the methylation level changes of two DNA methylation forms, m6A and m5C, of *E. coli* under different growth conditions were characterized [103]. Likewise, the methylation patterns within the *opvAB* regulatory region of *S. enterica* for *opvAB* expression regulation were characterized by SMRT sequencing [48]. These results provided insight into the DNA methylation control in bacteriophage resistance as well as adaptation under different environmental factors. In addition, SMRT and Nanopore sequencing empower researchers to detect different DNA methylation motifs and modified bases within novel genome sequences, along with the DNA methylase prediction based on REBASE, a comprehensive database providing experimentally validated DNA methylases including their source organisms and recognition sequences [104]. As of 9^th^ April 2025, the DNA methylation system of over 4900 organisms (including bacteria, archaea, eukaryotes, and viruses) from SMRT data has been recorded in REBASE [104]. From a future perspective, integrating the third-generation sequencing and REBASE database to characterize the prokaryotic DNA methylation systems and phase-variable DNA methylases may help overcome the challenges in pathogenic control due to phase variation under epigenetic control.

Meta-epigenomic analysis through third-generation sequencing may facilitate the study of ecosystem development. Usually, the microbial structure of a specific ecosystem can be characterized by meta-genomics; however, without support from other omics data, microbial communication cannot be estimated. Encouragingly, researchers can concurrently obtain the meta-genomic and meta-epigenomic data via third-generation sequencing. Given the close association of DNA methylation with gene expression [7, 8], the utilization of third-generation sequencing may provide a more efficient and cost-effective approach to estimating microbial communication and even the host–microbe interaction of an ecosystem. For example, several studies have investigated the meta-epigenomic patterns of the gut [97, 105], freshwater [106], and ocean samples [24, 25] using SMRT or Nanopore sequencing. Considering the prokaryotic RM system in host genome defence against viruses, the study of the viral and prokaryotic epigenome systems in the ecosystem may enhance our understanding of the prokaryotic adaptation and virus-prokaryote co-evolutionary history [24, 25]. However, integrating other omics data, such as meta-transcriptomic or metabolic data, is still recommended to validate the dynamic interaction of an ecosystem.

Generally, artificial intelligence (AI) approaches (e.g. machine learning and deep learning) utilize various algorithms to train models for DNA methylation detection, which are ready for user application once developed. Integrating model-based or AI strategies has enhanced the efficiency and scale of DNA methylation detection and analysis using third-generation sequencing. For instance, the employment of a convolutional neural network for SMRT data model training predicted an m5C profile with a remarkable 99% correlation to bisulfite sequencing results without enzyme or chemical conversion [95]. In addition, statistical test strategies require both unmethylated and methylated datasets for DNA methylation identification using third-generation sequencing, increasing the demand for data storage resources. In comparison, model-based strategies can improve data storage efficiency for prokaryotic DNA methylation analysis, because unmethylated datasets are unnecessary for most users. However, while model-based approaches are efficient, their accuracy can vary due to different background factors in the training dataset (e.g. sequencing techniques and sequence contexts) [93, 97]. Therefore, while AI’s ability to detect complex patterns has led to the ability to characterize prokaryotic DNA methylation, such complex models are susceptible to contextual variation, and thus, care should be taken in their applications, specifically in the form of appropriate control samples and experimental designs.

Taking advantage of the capability of signal deviation detection based on the DNA structures, the third-generation sequencing technology also has the potential to detect various DNA modification forms beyond methylation. For example, DNA phosphorothioation (PT) is the substitution of non-bridging oxygen in the DNA sugar-phosphate backbone with sulphur. The PT modification was first identified in bacteria with *dnd* gene cluster [107] and has been shown to affect gene expression and interact with the RM system for bacterial genome defence [108], suggesting the potential regulatory role of PT modification in bacteria. Although there is no official detection model for DNA PT so far, studies have shown that both SMRT [109] and Nanopore [110] sequencing have DNA PT detection capability. Therefore, by combining with other omics data, the development of a DNA PT characterization model for third-generation sequencing could help reveal the comprehensive role of the DNA modification system in prokaryotic lifecycle and adaptation.

## Conclusion

DNA methylation regulates various biological activities, such as genome defence, gene regulation, and DNA repair in prokaryotes. Orphan and phase-variable RM system methylases modulate gene expression through various mechanisms, facilitating the adaptation of prokaryotes to changing environmental factors. The application of current sequencing techniques, such as PacBio’s SMRT and ONT’s Nanopore, allows the in-depth analysis of prokaryotic epigenetic systems. While these technologies are still under active development, they are opening up the possibility of easily accessible prokaryotic methylomes, something that was previously difficult to obtain.

## Data Availability

Data sharing is not applicable to this article because no datasets were generated.
